# Empty Beds in Nursing Homes Filled by Younger Individuals Following ACA Medicaid Eligibility Expansion

**DOI:** 10.1093/geroni/igaa057.585

**Published:** 2020-12-16

**Authors:** Ashley Ritter, Norma Coe, Salama Freed

**Affiliations:** University of Pennsylvania, Philadelphia, Pennsylvania, United States

## Abstract

National decline in nursing home occupancy rates coupled with expansion of Medicaid insurance eligibility under the Affordable Care Act (ACA) potentially create an opportunity for younger, low-income individuals to enter nursing homes following a hospitalization. Changes in the population of individuals using nursing homes could result in downstream consequences on facility payor mix, case severity index, and ultimately patient outcomes. This study measures the effect of ACA Medicaid eligibility expansion on the patient population using nursing homes, accounting for the nursing homes’ occupancy rate. Data were obtained from the publicly available national dataset, LTCfocus (2009-2016). Difference in differences estimation with time and state fixed effects was utilized to examine the effect of ACA Medicaid eligibility expansion on two outcomes, 1) average age in years of residents as of April 1 and 2) the proportion of individuals covered by Medicaid insurance at the facility level. Results show facilities with pre-ACA occupancy rates between 40% and 50% demonstrated the largest decrease in average age by year three, 1.32 years [95% CI: -2.257, -0.385]. Facilities with a pre-ACA occupancy rate of 60-70% demonstrated the largest increase in the proportion of individuals covered by Medicaid in year one, a 5.5 percentage point increase [95% CI: 0.009, 0.102]. In summary, Medicaid expansion under the ACA resulted in an increase in younger individuals and individuals covered by Medicaid using nursing homes, varying across pre-ACA occupancy rates. It remains to be studied if increased utilization of this high cost setting provides superior patient outcomes for these populations.

